# Genome-wide analysis of strand-specific transcription and DNA methylation in *Plasmodium falciparum* severe malaria

**DOI:** 10.3389/fmicb.2026.1846174

**Published:** 2026-06-29

**Authors:** Sukriti Gujarati, Bharat Raj Singal, Syamantak Majumder, Sanjay Kumar Kochar, Dhanpat Kumar Kochar, Ashis Das

**Affiliations:** 1 Department of Biological Sciences, Birla Institute of Technology and Science (BITS), Pilani, Rajasthan, India; 2 Department of Medicine, S.P. Medical College, Bikaner, Rajasthan, India

**Keywords:** CpG methylation, integrative epigenetics, malaria, nanopore sequencing, natural antisense transcripts, *plasmodium falciparum*

## Abstract

**Introduction:**

Gene expression and regulation are central to the survival and adaptation of *Plasmodium falciparum*. However, the interplay between transcriptional activity, Natural Antisense Transcripts (NATs), and DNA methylation in clinical isolates associated with different malaria manifestations remains poorly understood.

**Methods:**

Here, we present an integrated, strand-specific transcriptome and methylome analysis of *P. falciparum* derived from patients with uncomplicated malaria (UNC), hepatic dysfunction (HD), and cerebral malaria (CM), using custom, strand-aware microarrays, and directional short-read RNA sequencing. We also performed long-read Nanopore sequencing to simultaneously identify 5-methylcytosine (5mC) and 5-hydroxymethylcytosine (5hmC) modifications in a subset of these isolates.

**Results:**

We identified widespread NATs expressed in distinct disease manifestations. Transcriptional regulation in *P. falciparum* appears to require an expression flux in which coordinated S-AS expression occurs in the nucleus, whereas mitochondrial genes exhibit a high functional demand with limited antisense control. NATs complementary to rRNA species reveal a potential regulatory role for rRNA stability in parasites. We report novel associations between mitochondrial respiration and disease severity. Despite globally low methylation levels, intragenic methylation is consistently associated with transcription, with the enrichment of differentially methylated events within exons.

**Discussion:**

Together, we provide the first integrated view of DNA methylation and global transcription in clinical isolates of *P. falciparum*, elucidating a multilayered regulatory mechanism of parasite adaptation in the host, paving the way for disease-specific, informed antimalarial approaches.

## Introduction

Malaria remains a major contributor to the global health burden of infectious diseases, with an estimated 282 million global cases in 2024—an increase of 9 million from 2023, driven largely by surges in the WHO East African regions (World malaria report, 2025). The most prevalent and pathogenic malaria parasite, accounting for more than 90% of malaria mortality, especially in the WHO African region, is *Plasmodium falciparum* (Mace et al., 2018). As the parasite progresses through its ~48-h intra-erythrocytic developmental cycle (IDC), it presents a range of clinical outcomes in the human host, depending on host microenvironmental factors and parasitaemia, often leading to disease severity (MacKintosh et al., 2004; Thomson-Luque et al., 2021). This leads to clinical symptoms being classified as uncomplicated (non-severe) to complicated (severe) malaria (Gilles, 2012; World Health Organization, 2015), accounting for more than half a million deaths every year globally (Taylor-Robinson et al., 2007). Uncomplicated malaria typically presents with non-specific symptoms such as fever, chills, headache, malaise, vomiting, and myalgia, without evidence of vital organ dysfunction. In contrast, severe malaria is defined as a clinically complicated progression of the disease with life-threatening symptoms, like cerebral malaria (coma or impaired consciousness), renal failure, respiratory distress syndrome, hypoglycemia, shock, jaundice, spontaneous hemorrhage, convulsions, and/or evidence of vital organ dysfunction (Omari and Garner, 2004; WHO guidelines for malaria, 2025).

Cerebral malaria remains the most lethal manifestation of severe malaria with a 15–25% fatality rate even with current first-line antimalarial therapy, with some survivors also experiencing long-term neurological impairment (Seydel et al., 2015). Hepatic dysfunction (HD) is another severe malaria manifestation associated with metabolic derangements, though typically with lower mortality than CM (Anand and Puri, 2005). While these complications are thought to arise from microvascular obstruction of capillaries by parasites during their IDC stage (Bernabeu and Smith, 2017), the parasite-intrinsic molecular mechanisms underlying these complications remain poorly defined. Transcriptome analysis of the *P. falciparum* culture strains during the IDC follows a rigid, highly regulated transcriptional cascade with the progression of infection (Bobowik et al., 2025; Bozdech et al., 2003). Therefore, it is rational to assume that differential gene expression in the parasites would be an important aspect, along with host microenvironmental factors, governing this heterogeneity of clinical manifestations (Tonkin-Hill et al., 2018; Wichers et al., 2021). Studies investigating the transcriptome from culture strains do not fully represent the *in vivo* environment or host-imposed pressures (Tonkin-Hill et al., 2018; Wichers et al., 2021; Daily et al., 2004; Alvarez et al., 2021; Karikari et al., 2021). As a result, molecular features unique to specific severe manifestations of malaria remain largely unexplored. Moreover, studies comparing *in vivo* and *in vitro* parasite transcriptomes have found considerable differences in the parasite gene expression profile (Daily et al., 2004).

Another potential factor influencing gene expression is DNA methylation, which has been largely overlooked in previously published transcriptomic studies. Epigenetic modifications are increasingly recognized as mediators of transcriptional reprogramming, enabling phenotype switching, virulence adaptation, and developmental transitions (Mishra et al., 2011; Wang et al., 2021; Gao et al., 2012; Hollin et al., 2023; Katoh et al., 2006). Due to the challenges imposed by the unusually high AT content of the *P. falciparum* genome, epigenetic studies investigating the cytosine base modifications are limited. Although cytosine DNA methylation levels in *P. falciparum* have been reported to be genomically sparse (Ponts et al., 2013), their *in vivo* levels under disease-specific conditions remain undefined (Nardella et al., 2020). The effect of DNA methylation is context-dependent: it is a gene-repressive mark in the promoter region; when occurring in the gene body, it is positively correlated with gene expression in humans and other organisms (Li, 2021; Yang et al., 2014; Lim and Maher, 2010). The relationship between methylation and gene expression in parasites remains unclear and appears variable across species and genomic contexts (Jjingo et al., 2012; Hammam et al., 2020; Lucky et al., 2023). To date, no study has examined the correlation between locus-specific DNA methylation and gene expression in clinical isolates representing distinct malaria severities.

In addition to modulation by DNA methylation, transcription from the opposite DNA strand (i.e., the antisense strand), gives rise to Natural Antisense Transcripts (NATs), adding another layer of complexity to parasite gene expression and regulation. NATs are pervasive in the *P. falciparum* genome and have been assigned roles in gene expression regulation, including the degradation of corresponding sense transcripts or the silencing of genes at the chromatin level (Faghihi and Wahlestedt, 2009; Gunasekera et al., 2004; López-Barragán et al., 2011). While a study on the *P. falciparum* 3D7 and NF54 strains reports that var antisense long non-coding RNA (lncRNA) influences gene transcriptional regulation (Jing et al., 2018), another study reports no consistent influence of antisense transcripts on protein levels (Siegel et al., 2014). At the gene level, NATs can regulate *P. falciparum* virulence gene expression (Amit-Avraham et al., 2015; Epp et al., 2009). Thus, the extent of NAT expression across different disease severities remains unresolved, especially *in vivo*, where regulatory demands differ from those in cultured parasites (Subudhi et al., 2014; Vembar et al., 2014).

Antisense transcription has also been mechanistically linked to epigenetic regulation (Duraisingh and Skillman, 2018). Studies across mammals, plants, fungi, and protozoan parasites increasingly suggest that DNA methylation frequently acts in coordination with non-coding RNAs, indicating that multiple regulatory layers collectively shape the transcriptional states associated with pathological conditions (Huang et al., 2022; Wanowska et al., 2018; Urquiaga et al., 2021; Hoo et al., 2016). Collectively, this supports a unified regulatory model in which differing host microenvironmental pressures may induce epigenetic and transcriptional plasticity in *P. falciparum*, ultimately influencing disease severity and clinical outcomes of malaria.

We hypothesize that clinically distinct manifestations of malaria may result from the combined regulatory mechanisms within the parasites. It is, therefore, imperative to simultaneously investigate DNA methylation, NATs expression, and mRNA transcription in parasites showing different disease manifestations. To address these gaps, we performed comprehensive microarray-based transcriptome profiling of parasites isolated from 22 adult malaria patients across three clinically distinct groups: uncomplicated malaria (UNC, *n* = 6), cerebral malaria (CM, *n* = 4), and hepatic dysfunction (HD, *n* = 12; Gilles, 2012; World Health Organization, 2015) and investigated the profile of both sense and NATs from nuclear, mitochondrial, and apicoplast genomes. To validate the transcriptome, we pooled RNA from a subset of samples to represent the two distinct disease groups, ‘Uncomplicated pool’ and ‘Complicated pool’ (referred hereafter), and performed strand-specific short-read RNA sequencing (Assefa et al., 2020). To investigate potential epigenetic associations, we further assessed DNA methylation levels in CpG context in a subset of isolates using long-read Nanopore sequencing (Li, 2021). By analysing individual patient isolates from distinct severe disease manifestations, this study provides novel insights into the molecular mechanisms underlying *P. falciparum* infection in humans.

## Results

### Detection of widespread NATs from the nucleus as well as organellar genomes

**Figure 1:**
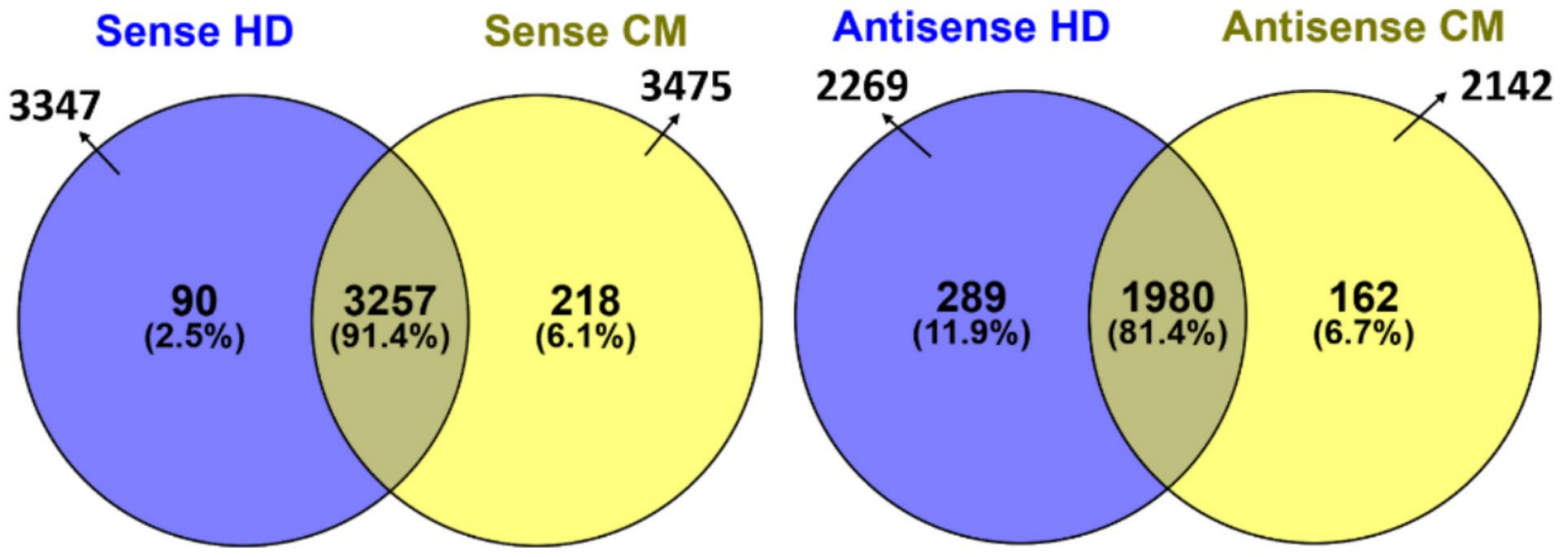
Number of genes exhibiting sense and antisense transcripts (NATs) in HD and CM, supported by at least three probes per gene. Overlapping regions indicate genes common to both conditions. Only probes detecting transcripts in at least 40% of samples within each disease group were included in the analysis. For CM analysis, samples included CM isolates (*n* = 4) and uncomplicated controls (UNC, *n* = 6); for HD analysis, samples included HD isolates (*n* = 12) and the same UNC cohort (*n* = 6). Arrows indicate the total number of genes in each disease cohort, with a minimum of 3 probes per gene.

To assess global sense and antisense transcription in *P. falciparum*, a total of 5,561 unique genes encoded from the nucleus, mitochondria, and apicoplast genome of *P. falciparum* were represented in the custom-designed 60 k microarray in both sense and antisense orientation, out of which more than 60% of sense and antisense ORFs were detected 2-fold against the background by at least 1 probe in both the disease cohorts. To further increase the confidence of detection in gene expression, three stringent filtering criteria were applied: (i) Only the probes that have detected transcripts in at least 40% of samples within individual disease groups were considered. For the CM analysis, the dataset comprised CM isolates (*n* = 4) along with uncomplicated control isolates (UNC, *n* = 6). Similarly, the HD analysis included HD isolates (*n* = 12) and the same shared UNC cohort (*n* = 6), (ii) probes for the same transcript with correlated expression (PPC > =0.8) were selected (as described in *Methods*), (iii) a particular transcript was detected with a minimum of 3 probes. The remaining transcripts were eliminated from the analysis. A total of 3,347 sense and 2,269 NATs in HD were detected, while 3,475 sense and 2,142 NATs in CM were seen with a minimum of 3 probes/gene. ~91% of sense transcripts and ~81% of NATs were detected with at least 3 probes across both CM and HD (Figure 1).

To estimate transcription coverage of nuclear and organellar DNA, the distribution of expressed transcripts across each chromosome, as well as the mitochondrial and apicoplast genomes, was evaluated. Less than 10% of sense transcription was detected in the apicoplast genome, whereas it ranged from ~40 to 65.45% in the chromosomal and mitochondrial genomes. More than 25% of genes on each chromosome and in the mitochondrial genome showed antisense transcription in both disease conditions. The apicoplast genome showed relatively low antisense transcription, a trend similar to that observed for sense transcription (Supplementary Figure S1).

### Expression trend of sense–antisense transcript pairs

**Figure 2:**
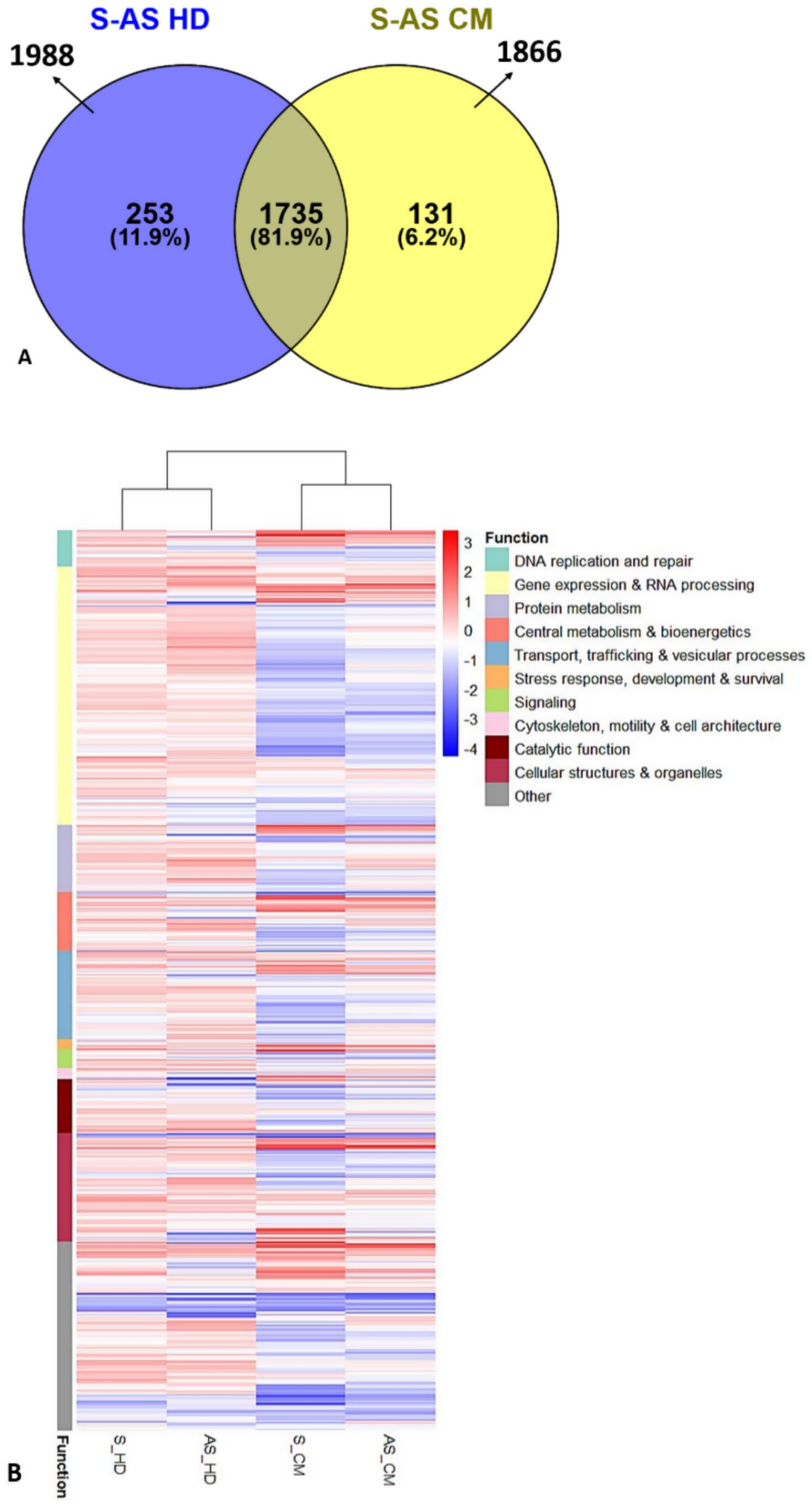
Shared sense–NAT transcript pairs (S-AS) across cerebral malaria (CM) and Hepatic dysfunction. **(A)** Venn Diagram showing the overlap of sense-NATs expressed across both disease manifestations (HD; blue, *n* = 1,988 genes | CM; yellow, *n* = 1,866 genes). Overlapping region (*n* = 1,735, 81.9%) represents pairs expressed in both CM and HD. Non-overlapping regions represent condition-specific Sense-NATs transcript pairs. A pair was considered detected if both the sense and antisense transcripts exceeded the detection threshold (40% of samples within a condition) with 3 or more probes. **(B)** Heatmap representing log_2_-transformed average expression values of 1,735 sense-NAT pairs common across both disease cohorts. Each row represents a gene, grouped by its functional categories on the left. Each column represents the four distinct conditions (AS_CM: antisense transcripts in CM, S_CM: sense transcripts in CM, AS_HD: antisense transcripts in HD, S_HD: sense transcripts in HD), arranged by hierarchical clustering (average linkage, Euclidean distance). The color scale shows log_2_ fold change values.

**Figure 3:**
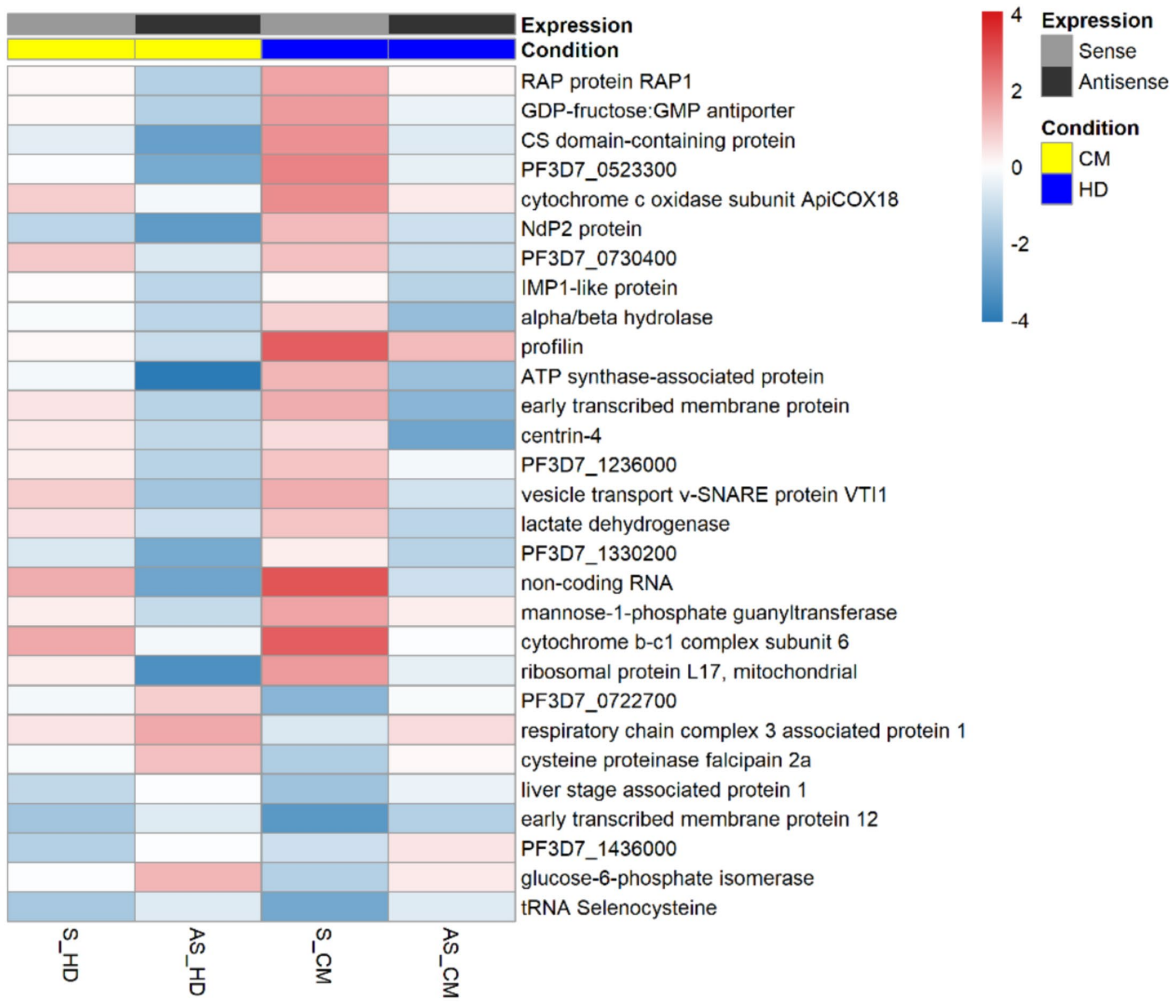
Heatmap representing average log₂-transformed expression values of 29 genes with discordant expression of sense-NATs across the two disease cohorts. Discordance was defined as a statistically significant inverse relationship (≥2-fold difference) between sense and NAT expression confirmed by a one-sample *t*-test (*p* ≤ 0.05). Top annotation bars indicate transcript type (grey = sense, black = antisense) and disease condition (yellow = CM, blue = HD). Color scale represents relative expression levels (blue = low, red = high); HD: hepatic dysfunction; CM: cerebral malaria; S: sense expression; AS: antisense expression.

A total of 1988 sense-antisense (S-AS) transcript pairs were expressed in the hepatic dysfunction (HD) group, and 1866 pairs were expressed in the cerebral malaria (CM) group. A total of 1,735 genes (81.9%) expressed sense-NAT pairs (S-AS) in the two disease cohorts, while 131 and 253 S-AS pairs showed disease-specific expression, respectively (Figure 2A). To visualize the expression patterns of the S-AS pairs for these 1,735 genes across the two disease groups, we generated a heatmap of their average fold-change values (Figure 2B). S–AS pairs from diverse functional categories exhibit comparable expression patterns across CM and HD, with more than 75% of the gene pairs showing significant positive correlation in both CM (*n* = 1,636, *ρ* = 0.69) and HD (*n* = 1,530, ρ = 0.52) disease cohorts. In contrast, only 29 genes showed a strong discordant expression pattern between their sense and antisense transcripts across both HD and CM (Figure 3).

### Differential gene expression across the two disease cohorts

**Figure 4:**
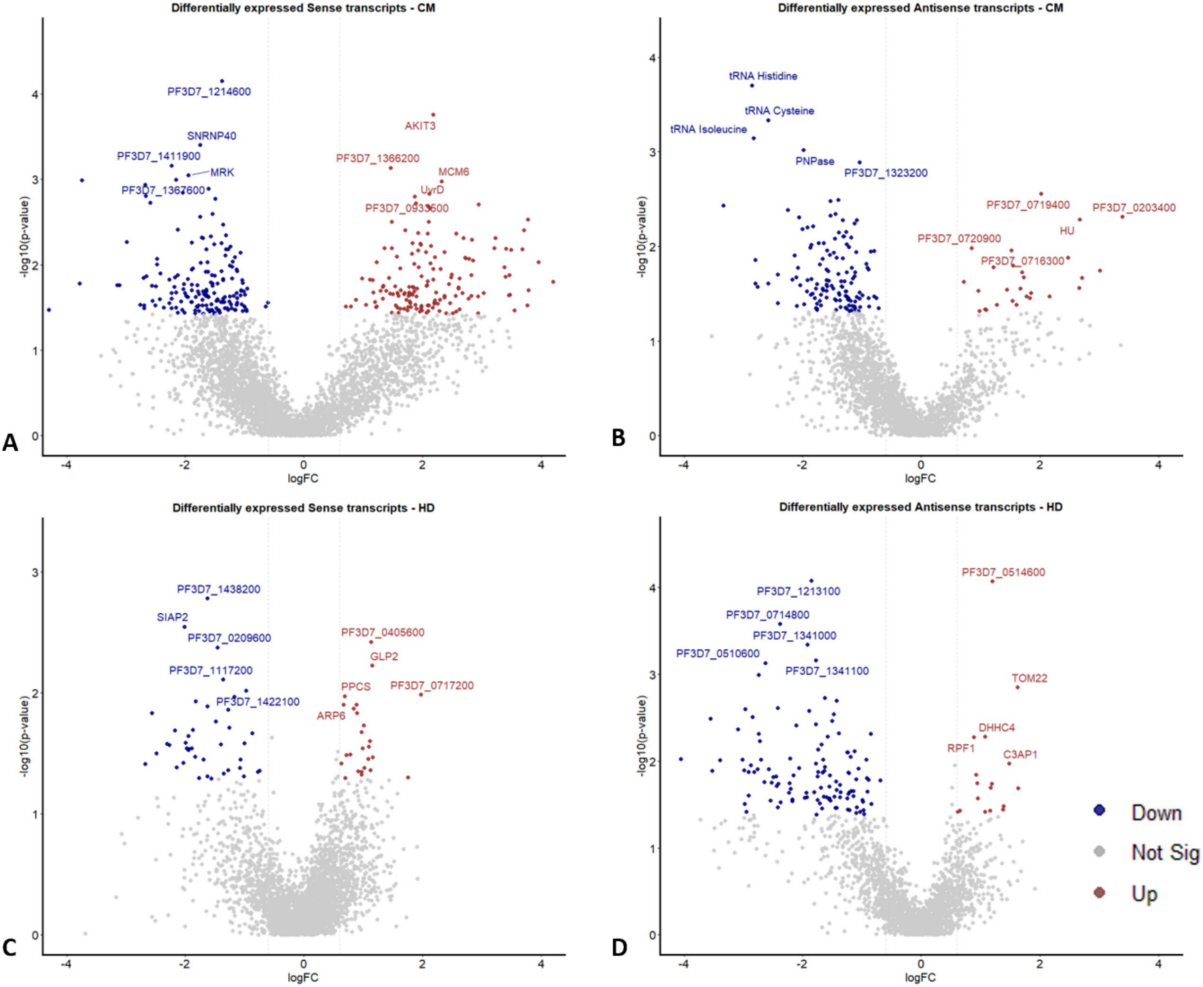
**(A-D)** Volcano plot of differentially expressed genes identified between the control group (UNC), and **(A)** Sense transcripts in cerebral malaria, **(B)** antisense transcripts in cerebral malaria, **(C)** Sense transcripts in hepatic dysfunction, **(D)** antisense transcripts in hepatic dysfunction. The X-axis indicates the log-transformed mean expression for each gene, and the Y-axis indicates the *p*-values. Upregulated transcripts are shown in red; downregulated in blue; non-significant in grey. Top 10 genes with FDR < 0.05 are labelled on the plot.

To evaluate disease-specific trends across CM and HD, differentially expressed sense and NATs (log2 fold change ± 0.6 and *p*-value ≤ 0.05) within each disease group were identified. The *p*-values were adjusted using Benjamini-Hochberg correction (Supplementary Dataset 2). The CM disease cohort showed 198 upregulated and 220 downregulated sense genes. The HD disease cohort had 23 upregulated sense, 20 upregulated NATs, while 39 downregulated sense and 138 downregulated NATs, respectively (Figures 4A–D). Three genes from sense loci: PF3D7_0704400 (phosphoinositide-binding protein, putative), PF3D7_0721400 (rhoptry protein, putative), and PF3D7_1032600 (conserved protein, unknown function), and one gene from antisense loci (PF3D7_1309400, HORMA domain protein, putative) were upregulated across both manifestations.

### Expression trends from the organellar genome

Plasmodial organelles, mitochondria, and apicoplast have remained important and validated drug targets (van Dooren et al., 2005; Mukherjee and Sadhukhan, 2016; Greco et al., 2025; Goodman et al., 2017). The mitochondrial genome encodes 42 genes on a 6-kb chromosome, of which we could include only 20 ORFs in the array; the rest being too short for 60-mer probe design. Of these 20 ORFs, we captured transcripts from 18 ORFs in both sense and antisense orientations across both disease conditions (Supplementary Figure S2). The mitochondrial genome encodes three protein-coding genes: PF3D7_MIT01400 (cox3), PF3D7_MIT02100 (cox1), and PF3D7_MIT02300 (*cytb*), which form the core of the respiratory electron transport chain in the plasmodial mitochondria, and their expression has been reported in previously published studies (Wichers et al., 2021; Llinás et al., 2006; Pelle et al., 2015; Young et al., 2005). High expression of sense transcripts was identified for all three genes on the array, with relatively low levels of their corresponding NATs. This suggests enhanced demand for mitochondrial cytochrome subunits in severe malaria, and they may not be regulated by their NATs. Apart from the protein-coding loci, S-AS pairs were also detected for 15 rRNA fragments encoded by the mitochondrial genome. These ribosomal RNA fragments reportedly form the mitochondrial ribosome (mitoribosome), together with ribosomal proteins imported from the cytosol (Ling et al., 2020).

*P. falciparum* apicoplast harbors 67 genes, out of which 30 are protein-coding, and the rest encode for tRNA/rRNA. Our array could include only sense and antisense probes for 33 genes from apicoplast genomes, and the rest had to be excluded from the array design due to cross-hybridization or too-short gene sequences for 60-mer probe design. Of which, 2 apicoplast genes (PF3D7_API05900 and PF3D7_API01500) showed both sense and NAT expression across both disease cohorts at our analysis thresholds. Since the microarray provided only a limited representation of genes from the apicoplast genome, the detection of apicoplast-encoded transcripts was investigated from the RNAseq dataset. Sense transcripts for 4 apicoplast-encoded genes were detected: PF3D7_API01500 (RPL2), PF3D7_API02700 (RPS12), PF3D7_API02900 (TUFA), PF3D7_API03600 (ClpM). All detected transcripts showed relatively low expression in the complicated sequencing pool, and their corresponding NATs were not detected. To evaluate cross-platform consistency, these four apicoplast genes were examined in the microarray dataset. Specifically, RPL2 and TUFA were detected with >3 probes in both CM and HD cohorts, whereas RPS12 showed limited probe representation in CM but >3 probes in HD. ClpM was not detected on the microarray in either disease condition. Transcriptomic studies from culture and other strains of *P. falciparum* have also reported minimal expression of apicoplast transcripts from asexual stages of infection (Bozdech et al., 2003; Jing et al., 2018; Lim and Maher, 2010; Otto et al., 2010; Chappell et al., 2020; Shaw et al., 2022; Toenhake et al., 2018).

### Validation of gene expression data through RNA sequencing

To independently validate the microarray findings, strand-specific RNA sequencing was performed on pooled RNA derived from isolates representing each clinical group. Following initial quality control and filtering, >93% of the bases exceeded Q30 scores in both pools. The processed, host-depleted reads from the two sequencing pools resulted in parasite alignment rates of 85.02% for the uncomplicated pool and 78.77% for the complicated pool.

At the read level, RNA-seq detected 4,549 sense transcripts and 2,643 NATs across both pools (Supplementary Dataset 3). Comparison of gene expression estimates between microarray and RNA-seq platforms revealed strong concordance. Sense transcript expression showed a strong positive correlation (*ρ* = 0.82, *p* < 2.2 × 10^−16^) while antisense transcripts exhibited a moderately positive yet statistically significant correlation (ρ = 0.52, *p* < 2.2 × 10^−16^), indicating overall agreement between the two methodologies (Supplementary Figure S3).

Transcript assembly and classification using StringTie, followed by gffcompare, identified 218 NATs in the uncomplicated pool and 112 in the complicated sequencing pool, of which 33 were shared between both disease manifestations (Supplementary Dataset 3). Gene Ontology enrichment analysis of these shared StringTie-assembled antisense transcripts revealed significant enrichment of the biological process of *protein palmitoylation* (Supplementary Figure S4). To further characterize these antisense transcripts, we examined their length distribution. Across both sample pools, antisense transcripts exhibited shorter transcript length, with the median length of 576 bp in the uncomplicated pool and 492 bp in the complicated pool (Supplementary Figure S5). A summary of transcript classification based on structural relationships to the reference annotation is provided in Supplementary Table S1.

### Gene ontology enrichment analysis of DEGs

GO enrichment analysis of upregulated sense transcripts in the cerebral malaria cohort revealed significant associations with pathways related to *DNA replication*, and biological processes of *mitotic nuclear division*, *chromosome organization*, *signal transduction*, and *cell cycle process*. Additionally, terms related to *DNA repair* were enriched. Pathways linked to mitochondrial function and cellular respiration, such as *the respiratory electron transport chain* and *Citrate cycle (TCA cycle)*, were also significantly enriched. In contrast, the number of upregulated sense transcripts in the HD cohort was comparatively lower and did not show significant enrichment for any biological processes. Genes with upregulated NATs in CM showed significant enrichment for GO terms related to *chromosome organization, sequence-specific DNA binding*, and *protein-DNA complex*. For upregulated NATs in HD, the top enriched GO terms were *lipoprotein metabolic process, S-acyltransferase activity,* and *protein-cysteine S-palmitoyltransferase activity*. Comparative analysis of downregulated sense and antisense transcripts between HD and CM cohorts revealed common enrichment terms such as *triplet codon-amino acid adaptor activity*, *mRNA binding*, and the KEGG pathway - *Aminoacyl-tRNA biosynthesis*. Disease-specific biological processes identified with downregulated NATs in HD include *ketone biosynthetic and metabolic process* and *mRNA processing* via *RNA splicing* in CM. A complete list of GO terms for all DEG sets is given in Supplementary Dataset 4.

### Global CpG methylation patterns and their association with gene expression

**Figure 5:**
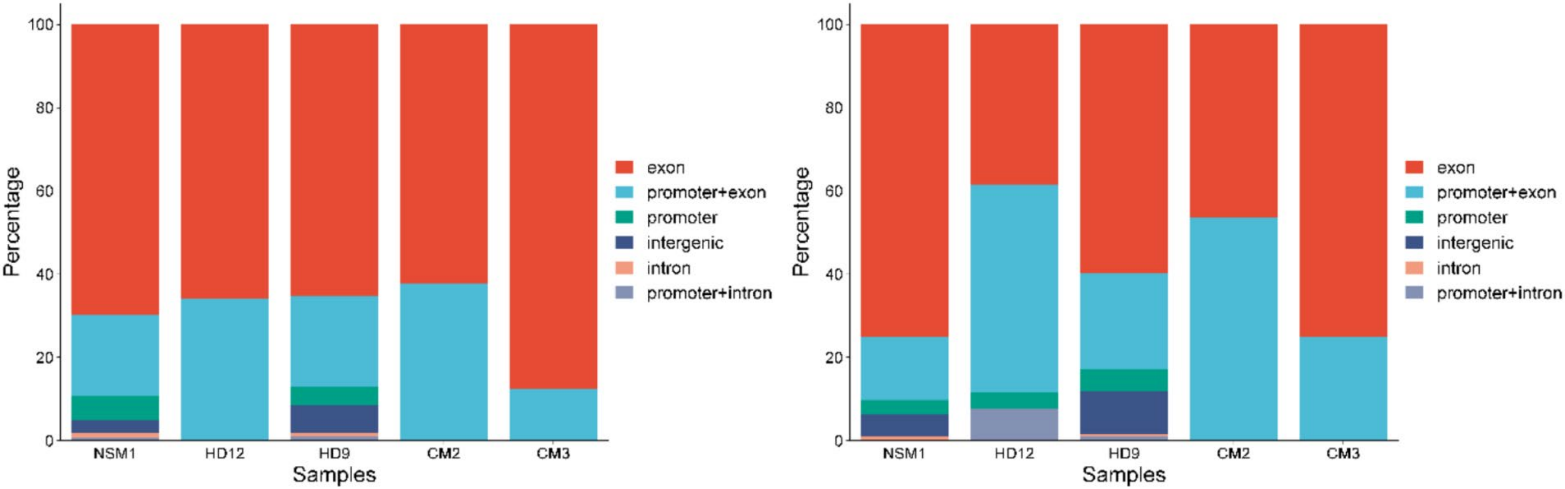
Proportion of CpG sites with 5mC (left) and 5hmC (right) modification (minimum read coverage >=5 and fraction of modified reads >=20%) that overlap annotated genomic features across the five sequenced isolates.

Genome-wide DNA methylome profiling revealed comparable levels of both 5mC and 5hmC across all sequenced isolates (Table 1). Global 5mC proportions ranged from 0.027 to 0.058 across samples, whereas global 5hmC proportions ranged from 0.081 to 0.152. Across all isolates, the global abundance of 5hmC was approximately 2-fold higher than that of 5mC, despite variability in sequencing depth, CpG site coverage, and clinical manifestations. Across all biological replicates, both 5mC and 5hmC modifications were predominantly mapped within exonic regions (>60%), with smaller proportions mapped to promoter, intronic, and intergenic regions (Figure 5).

**Table 1:** Genome-wide sequencing and CpG methylation statistics across all sequenced isolates.

Sample	CM2	CM3	HD12	HD9	NSM
Manifestation	Cerebral malaria	Cerebral malaria	Hepatic dysfunction	Hepatic dysfunction	UNC
Reads	3,61,473	67,724	9,87,657	2,77,560	5,37,334
Mean depth	0.24x	0.11x	0.7x	2.06x	5.53x
GC%	20.43%	19.07%	20.96%	12.01%	21.64%
Total CpG sites sequenced	39,492	23,136	104,240	206,572	283,984
Proportion of 5mC modified sites	0.055	0.05	0.052	0.027	0.031
95% CI (5mC)	0.052–0.058	0.046–0.054	0.051–0.054	0.026–0.028	0.030–0.031
Proportion of 5hmC modified sites	0.109	0.152	0.081	0.084	0.152
95% CI (5hmC)	0.105–0.113	0.146–0.159	0.079–0.082	0.083–0.086	0.151–0.153

Although most modified CpG sites were sample-specific, 53 CpG sites were shared across at least 3 isolates, of which 6 are distinct to the severe samples. All shared sites were localized within the exons of ribosomal RNA-encoding genes in the nucleus (PF3D7_0112700, PF3D7_0531600, PF3D7_0532000, PF3D7_0725600, PF3D7_0726000, PF3D7_1148640, PF3D7_1371300), mitochondria (PF3D7_MIT04000), and apicoplast (PF3D7_API05700, PF3D7_API05900). Given the tandem arrangement of rDNA loci, several of these sites also mapped to the upstream/downstream-regulatory region of neighbouring rRNA units.

**Figure 6:**
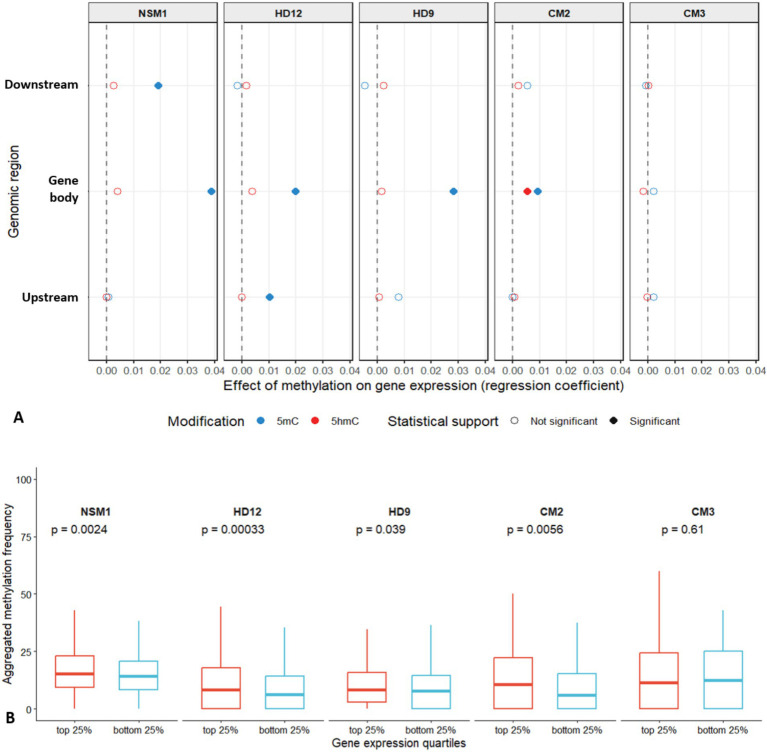
Relationship between CpG methylation and gene expression across sequenced isolates **(A)** Regression coefficients describing the association between CpG methylation and gene expression across genomic features. Gene expression was quantified as mean probe signal intensity (log_−_transformed) from microarray data. For each gene, aggregated methylation frequency was computed across three genomic regions (2-kb upstream, promoter-like, gene body, 2-kb downstream) separately for 5mC (blue) and 5hmC (red). Filled circles: statistically significant association (*p* < 0.05), open circles: not significant. Each panel represents one sequenced isolate. **(B)** The expression of genes in the top 25% and bottom 25% expression quartiles on the microarray is displayed as a box plot, along with the aggregated methylation frequency for each gene in each individual isolate sequenced. Gene expression quartiles were assigned based on mean probe signal intensity (log_−_transformed) from microarray data. Aggregated CpG methylation frequency per gene was calculated by normalizing per-site read depth by gene length. *p-value* indicates the statistical significance of each comparison.

To investigate the relationship between DNA methylation and transcription, gene expression was modelled as a function of methylation for both modification types and distinct genomic regions (gene body, 2-kb upstream, and 2-kb downstream) using linear regression. Gene body methylation consistently showed positive regression coefficients (Figure 6A). In contrast, methylation in upstream and downstream regulatory regions exhibited variable associations with gene expression. Given the significant association with gene-body methylation, the aggregated intragenic methylation frequency was compared between genes in the top and bottom 25% of expression percentiles based on microarray signal intensity. Across 4 out of 5 samples, genes in the top expression quartile exhibited a significant increase in aggregated methylation frequency compared with the bottom quartile (Figure 6B). The initial correlation analysis was performed on genes meeting minimal coverage criteria (as mentioned in methods—defined as ≥25% gene length covered by sequencing and the presence of at least three CpG sites per gene) without imposing a read-depth threshold. To assess the robustness of the observed association, the analysis was repeated with progressively higher mean gene-depth cut-offs (3×, 5×, 7×, and 10×). The positive association between gene-body methylation and expression remained consistent across these thresholds (Figure S6). To further assess the robustness of this association, the same analysis was performed using TPM values from RNA sequencing data generated from pooled clinical samples with similar disease manifestations. Highly expressed genes (by categorizing them into top and bottom 25% TPM quartiles) exhibited elevated intragenic methylation frequencies, even though the RNA-seq samples were not exact biological replicates of the methylation cohort (Supplementary Figure S7).

### Identification of differentially methylated regions

MethylLasso was used to segment genome-wide methylation profiles into 2 categories: Methylated domains, comprising UMR-DMV and PMD, identified independently in each isolate; and DMRs identified relative to the uncomplicated clinical isolate as the control. The 5mC methylome was dominated by UMR-DMV-like domains across all isolates. Only a single PMD was identified in one HD isolate. In contrast, analysis of 5hmC events identified a variable number of PMDs across isolates; however, their relative abundance to UMR-DMVs was low across all replicates. Because MethyLasso performs genome-wide segmentation independently for each sample, the resulting domain lengths can vary between samples. For cross-sample comparison, a custom script was used to identify genomic loci that show consistent domain classification by MethyLasso in at least 3 of the 5 isolates analysed. Using this approach, 46 regions were identified corresponding to DNA methylation valleys for 5mC modification. For 5hmC, 15 DMVs and 2 PMDs were shared by at least 3 of the 5 sequenced isolates (Supplementary Datasheet 5).

Across the samples, a total of 69 significant DMRs (methylation difference > = 25% and FDR < 0.1) were identified for 5hmC modification, while 1 DMR was identified for 5mC. Of these, 9 were hypermethylated, while 61 were hypomethylated relative to the uncomplicated malaria isolate (Supplementary Datasheet 5). Since the majority of these DMRs were annotated to exons, differential methylation was investigated in relation to gene expression overlapping these loci. All hypomethylated DMR-associated genes were found within the lowest 25% expression percentile.

## Discussion

In the current study, strand-specific microarrays and directional, short-read RNA sequencing was employed to assess genome-wide and organelle-wide transcription of both sense and NATs in *P. falciparum* clinical isolates. Due to limited RNA availability from field samples, RNA samples from similar manifestations were pooled and sequenced while maintaining adequate sequencing depth for downstream analysis (Assefa et al., 2020). Microarray results revealed that over 60% of genes expressed both sense and NATs across all clinical samples, and RNA-seq detected approximately 55% of genes expressing S-AS pairs. The concordance observed across two independent, strand-aware experimental techniques indicates NATs as a fundamental and widespread feature of gene regulation in malaria (Jing et al., 2018; Broadbent et al., 2015).

The relatively low transcription from the apicoplast genome compared to the mitochondrial and nuclear genomes likely reflects distinct regulatory mechanisms governing plastidial transcription. Upon comparing the expression analyses of apicoplast-encoded genes with published stage-specific and time-point transcriptomic studies on both clinical and laboratory strains of *P. falciparum*, similar findings have been reported for early IDC stages of the parasite (Bozdech et al., 2003; Wichers et al., 2021; Toenhake et al., 2018; Nisbet and McKenzie, 2016). Plastidial protein synthesis is generally reported to be synchronized with organellar maturation occurring primarily in the second half of the IDC (Wichers et al., 2021). Additionally, while antisense transcripts from apicoplast regions have been detected using techniques such as DAFT-seq, their abundance remains relatively low compared to nuclear NATs (Chappell et al., 2020; Nisbet and McKenzie, 2016; Nisbet et al., 2016). The minimal antisense expression observed in our study might be due to relatively low transcription of NATs in the apicoplast genome or experimental limitations inherent to microarray and short-read RNA-sequencing in capturing low-abundance organellar transcripts.

Our findings from the mitochondrial organelle, however, indicate elevated expression of protein-coding genes—*cox1*, *cytb*, and *cox3*, complemented with lower levels of corresponding NATs, suggesting an active ETC cycle within asexual blood-stage parasites under severe malaria. Previous reports on distinct physiological states in *P. falciparum* have established the use of alternative carbon sources and active respiration in response to variable microenvironmental oxygen and substrate levels *in vivo* (Daily et al., 2007). The enrichment of biological processes, such as the *respiratory electron transport chain*, associated with upregulated sense transcripts in CM further supports this hypothesis (Gujarati et al., 2026).

The mitochondrial genome of *Plasmodium* is also unusual due to its highly fragmented ribosomal RNA (rRNA) genes. Instead of a single, continuous rRNA gene, the parasite’s mitochondrial genome encodes numerous small rRNA fragments (around 20–200 bases) intermixed with protein-coding genes on both DNA strands. The rRNA fragments are essential to form the mitochondrial ribosome, together with ribosomal proteins imported from the cytoplasm, and have been proposed as an antimalarial drug target (Ling et al., 2020). Of the 17 rRNA genes represented on the array, we detected expression of NATs from 15 genes across all replicates in the current study. NATs for rRNA species have recently been reported to control rRNA stability during stress or apoptosis in *Leishmania infantum* (Padmanabhan et al., 2012). While this raises the possibility of a functionally analogous mechanism in Apicomplexan parasites, further experimental validation of NATs associated with mitochondrial rRNA fragments will be required in the context of this hypothesis in *Plasmodium*. When considered alongside the adaptive metabolic shifts reported in severe malaria, as observed by the elevated expression of key respiratory components, our data point towards an active mitochondrial transcriptional landscape that supports parasite survival under severe disease-associated stress conditions in asexual parasite stages (Evers et al., 2021; Van Dooren et al., 2006).

Genome-wide surveys in yeast, plants, mammals, and cancer cell lines have shown that ~30–40% of S-AS pairs exhibit positive correlation rather than the classical discordant expression pattern (Balbin et al., 2015; Das et al., 2024). In our study, more than 50% of S-AS pairs show concordant expression, as independently observed in both disease cohorts. A plausible mechanism for the observed positive S-AS co-expression is the presence of shared cis-regulatory elements, such as bidirectional promoters that drive transcription in both directions (Das et al., 2024; Trinklein et al., 2004). The compact genome architecture of *P. falciparum* is particularly conducive for efficient utilization of bidirectional promoter elements to drive transcription of the antisense transcript of the upstream gene, along with the sense transcript of the downstream gene (Boopathi et al., 2013). To investigate this relationship of NAT transcription influenced by its downstream sense neighbour, we examined expression of the NAT–S gene pairs arranged either on the same strand (tail-to-head orientation, no. of genes: 1,086 in CM and 1,052 in HD) or on opposite strand (tail-to-tail orientation, no. of genes: 1,370 in CM and 1,258 in HD). Across both disease cohorts, we observed significant but weak associations between NAT expression and its downstream sense neighbours arranged in either orientation, supporting a model in which shared regulatory elements contributing to coordinated transcriptional activity might be a gene-specific phenomenon (Jing et al., 2018). This co-expression of NAT-sense gene pairs accounted for only 45–49% of the detected NATs, indicating that antisense transcription may, in part, reflect local promoter architecture, independent of neighboring mRNA transcription. However, the observed concordant expression does not imply direct regulatory interactions between transcripts and may also arise due to overlapping transcriptional units, run-through transcription, chromatin-level effects, or alternative regulatory mechanisms.

In addition to broadly concordant S-AS pairs, we also identified severe disease-specific S-AS pairs that were negatively regulated across both CM and HD, including important parasite-specific genes such as ETRAMP (PF3D7_0936100), CEN-4 (PF3D7_1105500), VTI1 (PF3D7_1236000), and ApiCOX18 (PF3D7_0523300). Proteins of the ETRAMP family are known to be widely expressed in the asexual stages of all *Plasmodium* species at the host-cell interface (Sindhe et al., 2019; Spielmann et al., 2003). This molecule plays an important role in transporting proteins out of the parasite to sustain its survival within the host, and this role could be enhanced in cases of severe disease. Cen-4 and PfRFC-5 play an active role during cell division by regulating DNA replication machinery (Voß et al., 2023; Cullmann et al., 1995; Jirage et al., 2010). The rate and efficiency of parasite replication are directly linked to the severity of malaria (McDonald and Merrick, 2022). The enrichment of processes such as cell cycle, mitotic nuclear division, chromosome segregation, and DNA replication was specifically associated with sense transcripts in cerebral malaria. We propose this as a consequence of cerebral sequestration and the difference in the microenvironments of cerebral malaria and liver-associated disease. Kupffer cells’ hyperactivation in the liver leads to active phagocytosis and faster reduction of parasite burden in comparison to cerebral malaria (Murthi et al., 2006; Nobes et al., 2002; Akide Ndunge et al., 2022; Chotivanich et al., 2000). The elevated energy demands might indicate the adaptive metabolic response in actively dividing parasite cells sequestered within the brain vasculature (Gujarati et al., 2026). In contrast, the hepatic microenvironment in malaria is characterized by systemic dyslipidemia resulting from hepatic dysfunction and Kupffer cell-mediated inflammation (Kluck et al., 2019). Elevated levels of NATs in our study were associated with specific GO terms related to *DHHC palmitoyltransferases* (PATs) and *protein palmitoylation*. Blood-stage parasites utilize host-derived fatty acids, and *P. falciparum* palmitoylation activity is dynamic, modifying proteins involved in parasite invasion, morphogenesis, and intracellular development (Santos et al., 2015; Jones et al., 2012; Wang et al., 2020). The antisense-mediated modulation of protein palmitoylation could be a context-specific regulatory response to the altered availability of palmitoyl-CoA substrates for parasite DHHC-PAT activity underlying hepatic dysfunction (Mitamura and Palacpac, 2003). Whether these NATs act through their interaction with sense counterparts, chromatin-level modifications, or protein interactions at the DHHC-PAT loci requires functional investigation (Zhang et al., 2020). However, these are observational speculations pointing towards disease-specific pathologies that cannot be determined from the current dataset, and rigorous functional validation will be required to test these hypotheses in a clinical setting with a larger sample size.

Beyond the NAT-mediated gene regulation, we also present here the base-resolved CpG methylation profile of clinical isolates of *P. falciparum* from distinct malaria manifestations. Though we intended to describe the genome-wide methylation of these isolates, the samples used in the study are field isolates of *P. falciparum*, which inherently have a much higher concentration of host DNA carryover, which is difficult to eliminate completely despite leucodepletion, resulting in lower sequencing depth for all samples. Therefore, to maintain data robustness, the methylation status of only the regions of the parasite genome sequenced with adequate coverage (>=5 reads) is used for all downstream analyses (Wei et al., 2017; Faulk, 2023).

Foundational studies investigating methylation in *P. falciparum* have characterized PfDNMT2/TRDMT1 (DNA methyltransferase 2/tRNA aspartic acid methyltransferase 1) as the primary methyltransferase enzyme responsible for selective methylation of tRNA (Gayathri Govindaraju et al., 2017; Goll et al., 2006; Jurkowski and Jeltsch, 2011; Erdmann and Picard, 2020). Subsequent parallel evidence of genome-wide cytosine methylation emerged from Ponts et al. (2013), who suggested *de novo* DNA methylation in the parasites, mediated by a single functional PfDNMT, raising the possibility of PfDNMT2’s substrate flexibility. However, the precise enzymatic mechanism of DNA methylation remains unresolved. Earlier studies employed diverse approaches, such as LC–MS, BS-seq, and oxBS-seq, to quantify cytosine DNA methylation. Each of these techniques has its own limitations, thus accounting for variable levels of cytosine modifications (~0.1–0.2% for 5mC, 0.5–0.9% for 5hmC, 0.2–0.4% for 5hmC-like base; Ponts et al., 2013; Lucky et al., 2023). While LC–MS offers high chemical specificity, it lacks the positional resolution provided by bisulfite-based methods (Lucky et al., 2023). Previous bisulfite-based studies used apicoplast as a non-methylated internal control for error modeling (Ponts et al., 2013). While this is a practical workaround for the AT-rich genome of *Plasmodium*, it assumes that any modification signal within the apicoplast genome is background noise. In our study, we observe apicoplast cytosine modifications across all samples (Supplementary Figure S7), suggesting that apicoplast may not be a suitable alternative as a non-methylated reference, especially in hypomethylated genomes like those of *Plasmodium*.

Despite lower sequencing depth, our Nanopore-derived methylation signals address these limitations and provide simultaneous locus-specific, base modifications from native DNA, complementing the prior studies (Hammam et al., 2020; Lucky et al., 2023; Huang et al., 2021). A substantial overlap was observed between the 5hmC-like CpG loci derived by Hammam et al. using oxBS-seq and the 5hmC-modified CpG sites sequenced in our Nanopore dataset (Supplementary Table S3). Differences in biological context also contribute to the variability observed among these studies and among different clinical isolates, as our samples are derived from patient isolates that experience unique, individual host-associated stresses and metabolic states, absent in WBC-sparse *in vitro* cultures (Lucky et al., 2023). The abundance of 5hmC modification may be attributed to the high oxidative stress imposed by the host microenvironment (Nardella et al., 2020; Hammam et al., 2020). The exact mechanistic basis of methylation and conversion from 5mC to 5hmC in *P. falciparum* remains a critical open question for the field. The possibility that *Plasmodium* employs non-canonical pathways contributing to cytosine methylation remains an important area for future investigation (Mahfouz, 2010).

We attempted to correlate the genome-wide methylation with the transcriptome. Gene body methylation was analysed separately, along with 2-kb upstream and downstream regulatory regions. To reduce gene-length–dependent bias, CpG content was normalized as CpG density per kb, yielding more comparable regression estimates across genes. While hydroxymethylation in both gene body and upstream-downstream flanking regions has previously been reported to correlate with higher gene expression in the *P. falciparum* 3D7 strain, those signals were largely contiguous with gene-body peaks (Hammam et al., 2020). Consistent with this, our analysis revealed the most stable association between gene expression and intragenic methylation. Upon examining this association using RNAseq data generated independently, although not all comparisons reached statistical significance, the direction of effect was largely concordant (Supplementary Figure S7), supporting the reproducibility of the transcriptomes captured by the two techniques and an overall positive association between gene-body methylation and transcription across distinct expression profiling approaches. While cytosine methylation in the gene body of active genes has been previously reported in eukaryotes (Yang et al., 2014; Zemach et al., 2010), the relationship with gene expression in the current study design has been demonstrated through correlational analysis. Integration of methylome data with nucleosome profile and chromatin accessibility from the same isolates will clarify the direct mechanistic link behind this relationship. However, such approaches would remain challenging in the case of clinical isolates.

Intragenic methylation of the rDNA loci, reproducible across isolates, might indicate these DNA modifications as a constitutive epigenetic feature of the parasite genome. Recent reports show that methylation of the ribosomal DNA promotes transcription of these genes by preventing enrichment of repressive histone modifications (Huang et al., 2021). Mapping of these methylated rDNA loci to their respective genes revealed stable expression of these genes across all samples, suggesting that methylation within these sites might be crucial in transcription of ribosomal RNA genes.

To further examine the DNA methylation pattern from individual CpG site-level to domain-level across the genome, we used the tool MethyLasso and unified domains that showed a similar methylation profile across isolates (Supplementary Dataset 5). The observation of a large number of UMR-DMV across all isolates and of limited site-specific, partially methylated events indicates that the *P. falciparum* genome is hypomethylated overall. Most DMR-associated genes encode conserved *Plasmodium* proteins with unknown function, and these could be interesting from the perspective of epigenetic modulation and regulation under severe disease stress, highlighting their need for further characterization.

Among the DMR-associated genes, a key regulator of parasite heat shock response (PF3D7_1342900, PfAP2-HS) was identified as hypomethylated in the HD isolate despite high expression on the microarray, suggesting potential disease-associated epigenetic modulation of parasite stress-response pathways (Tintó-Font et al., 2021). Although the majority of the significant DMRs identified were from HD isolates, their correlation with gene expression revealed that genes with intragenic hypomethylation were weakly expressed (Crawford et al., 2026; Shann et al., 2008; Hon et al., 2012). Although the number of genes intersecting with DMRs and detected simultaneously on the microarray is limited, this observation is consistent with the lower methylation frequency observed in the bottom 25% gene expression quartile on the array. Such an association in *Plasmodium* could lead to novel gene-specific targeted regulation and warrants future investigation (Reyser et al., 2024).

We acknowledge that increasing the cohort size and sequencing depth would further improve the resolution of subtle methylation differences across the parasite genome. In addition, the present analysis focuses on cytosine modifications in the CG context, and a comprehensive assessment of cytosine methylation’s influence on transcription would require future investigations that also include non-CG methylation. Future studies including healthy/uninfected individuals as controls would be of interest for comparing both host and parasite signatures across distinct disease severities. Clinical isolates often contain heterogeneous developmental stages of the parasite, which can influence both the transcriptome and the methylome. To address this, we estimated stage composition by correlating clinical expression profiles with the IDC reference transcriptome reported by Painter et al. (2018). Both CM and HD isolates were clustered within a small developmental stage window (late ring/early troph) with more than 75% of genes mapping to 20–32 h post-invasion (hpi), indicating broadly similar stage distributions across the three disease cohorts. However, stage heterogeneity *in vivo* may impact both transcription and methylation signatures of the parasites, and therefore remains a limiting factor in such studies.

Despite these caveats, our study highlights two complementary regulatory layers in clinical isolates of *P. falciparum* - cytosine methylation and antisense transcription, both of which are independently associated with gene expression and are known to collectively contribute to transcriptional plasticity (Faghihi and Wahlestedt, 2009; Hoo et al., 2016). This study identifies key parasite-intrinsic NATs as potential modulators of parasite adaptation and highlights mitochondrial pathways as a relevant target for severe malaria interventions. The field of parasite non-coding RNA biology remains largely descriptive (Broadbent et al., 2015). Our findings provide additional evidence for prioritizing candidate NATs as inferred from gene expression and enrichment analyses. Downstream functional experimental validation of these hypotheses remains crucial to establish their molecular associations and antimalarial potential. We further provide evidence of coordinated methylome-transcriptome events from field isolates. The direct, simultaneous profiling of both 5mC and 5hmC modifications from rare and limited clinical samples associated with severe malaria enables the capture of regulatory events unlikely to be observed in homogeneous *in vitro* culture systems. Strategies targeting compounds like bisubstrate inhibitors that reduce DNA methylation have already been used in *Plasmodium* (Nardella et al., 2020). The in vitro activity of quinazoline derivatives against DNMT inhibitors has been studied in *P. falciparum* and *P. berghei*-infected mice (Bouchut et al., 2019). This foundational dataset from the field is important for guiding the development of antiplasmodial epidrugs (Reyser et al., 2024; Coetzee et al., 2020; Vanheer et al., 2020) and significantly benefits the field by offering a promising frontier to combat increasing drug resistance in malaria-endemic countries.

## Materials and methods

### Sample processing

Venous blood samples from malaria-infected adult patients at SP Medical College, Bikaner, India, were collected after informed patient consent and written acceptance, in accordance with the hospital’s ethical guidelines. Four strict patient inclusion criteria were defined: (i) Any other concurrent illness or viral infection was ruled out at the point of collection, (ii) presence of blood stage parasite by slide microscopy, (iii) RDT positivity, (iv) molecular confirmation of mono-infection with *P. falciparum* was verified by 18S rRNA and 28S rRNA gene-based diagnostic PCR (Pakalapati et al., 2013; Pakalapati et al., 2013). As per the guidelines by WHO and consultation from the team of clinicians at the SP Medical College, the patients exhibiting malaria symptoms were divided into three disease groups: Uncomplicated (UNC, *n* = 6), Hepatic Dysfunction (HD, *n* = 12), and Cerebral Malaria (CM, *n* = 4; Gilles, 2012; World Health Organization, 2015). All the patient metadata is provided in Supplementary Sheet 1. The erythrocytes after separation on Histopaque gradient (Histopaque 1,077, Sigma Aldrich) were washed with 1x sterile PBS, lysed with TRI Reagent (T9424, Sigma-Aldrich), and transported for further processing to BITS Pilani.

Total DNA and RNA were extracted from all samples using standard procedures (Tri-Reagent, Sigma-Aldrich). The concentration and purity of extracted DNA and RNA were evaluated using a NanoDrop UV/Vis spectrophotometer (SimpliNano™ spectrophotometer, GEHealthcare) and Qubit 4 Fluorometer (Invitrogen). The integrity of the RNA was analysed on TapeStation (Agilent), while that of DNA was assessed on 0.8% agarose gels. Each of these samples was hybridized individually to an 8 × 60 k custom-designed microarray. RNA from a subset of these samples was pooled (two samples per pool) to create an ‘Uncomplicated pool’ and a ‘Complicated pool’, which were then sequenced on the Illumina platform for transcriptome validation using an orthogonal technique. A subset of samples (with sufficient input DNA concentration >1 μg) was used for methylome sequencing on the Nanopore platform (marked in Supplementary Dataset 1).

### Microarray design, labelling, and hybridization

Building on previously validated probe designs from our earlier printed *P. falciparum* microarrays—244 K and 15 K (Subudhi et al., 2014; Subudhi et al., 2016), a custom 8 × 60 K Agilent microarray was designed for the current transcriptomic analysis using reference strain *P. falciparum* 3D7v54 sourced from PlasmoDB (Aurrecoechea et al., 2009). During analysis, the genes were re-annotated using the reference genome PF3D7v64. The array covered 61,649 probes, representing 5,561 genes from 5,800 annotated transcripts of *P. falciparum* 3D7. 60-mer oligonucleotide probes were designed to capture transcripts in both sense and antisense orientations, with a maximum of 5 probes per ORF to enhance detection accuracy. To minimize non-specific hybridization, any probe with cross-hybridization potential was eliminated. The final design comprised 27,442 sense and 27,289 antisense oligonucleotide probes, representing genes from the nuclear (*n* = 5,508), mitochondrial (*n* = 20), and apicoplast (*n* = 33) genomes, along with 3,459 human genic and non-genic probes for quality control and host contamination assessment in patient isolates. Probes were also designed to represent genes belonging to the three important multigene families of *P. falciparum–*var (*n* = 94), rifin (*n* = 175), and stevor (*n* = 41; inclusive of pseudogenes). This multi-probe, custom microarray provides a well-curated probe set, ensuring high-sensitivity, strand-specific gene expression profiling in *P. falciparum*.

The microarray hybridization and scanning were performed at Genotypic Technology, Bengaluru, India, an Agilent-certified facility. RNA samples were labeled using the Agilent Quick-Amp labeling Kit (Part Number 5190-0442). Total RNA was reverse transcribed at 40 °C using an oligo-dT primer tagged to a T7 polymerase promoter and converted to double-stranded cDNA. Synthesized double-stranded cDNA was used as a template for cRNA generation. cRNA was generated by *in vitro* transcription, and the dye Cy3 CTP (Agilent) was incorporated during this step. The cDNA synthesis and in vitro transcription steps were carried out at 40 °C. Labeled cRNA was cleaned up using Qiagen RNeasy columns (Qiagen, Cat No: 74106), and quality was assessed for yields and specific activity using the Nanodrop ND-1000.

Fragmentation of labeled cRNA and hybridization were done using the Gene Expression Hybridization kit of Agilent Technologies (*In situ* Hybridization kit, Part Number 5190-0404). Hybridization was carried out in Agilent’s Surehyb Chambers at 65 °C for 16 h. The hybridized slides were washed with Agilent Gene Expression Wash Buffers (Part Number 5188-5327) and scanned with the Agilent Microarray Scanner (Part Number G2600D).

### Microarray data analysis

Raw data extraction from Images was obtained using Agilent Feature Extraction software. Feature-extracted raw data (2-fold above background signal) was analyzed using Agilent’s GeneSpring GX Software (GeneSpring-GX). Normalization of the data was done in GeneSpring GX using the 75th percentile shift method (Percentile shift normalization is a global normalization, where the locations of all the spot intensities in an array are adjusted. This normalization takes each column in an experiment independently and computes the percentile of the expression values for this array across all spots (where *n* ranges from 0 to 100, and *n* = 75 is the median). It subtracts this value from the expression value of each entity. For genes represented by multiple probes, we calculated the Pearson probe correlation (PPC) coefficient from the probe signal intensities. Only replicate probes showing high correlation (>=0.8) were considered for further analysis. The signal intensities of probes correlated in expression were averaged to convert the probe-based data to a gene-wise format. Fold change values were obtained by comparing test samples (CM and HD cohort) with the average of control samples (UNC cohort). The expression values for all severe isolates (in individual test groups—CM and HD) were averaged to obtain the final log-transformed expression value for both sense and antisense transcripts. Significant genes (*p*val ≤ 0.05) differentially expressed with a fold change of ±0.6 (log2) in the severe disease cohort (CM and HD individually) relative to the control sample group (UNC) were identified.

### Functional and pathway analysis of DEGs

Gene Ontology enrichment analysis of the differentially expressed genes was performed using DAVID.^1^ We separately analysed eight distinct gene lists: Upregulated sense genes in HD (UP-S-HD), downregulated sense genes in HD (DOWN-S-HD), Upregulated sense genes in CM (UP-S-CM), downregulated sense genes in CM (DOWN-S-CM), Upregulated antisense genes in HD (UP-AS-HD), downregulated antisense genes in HD (DOWN-AS-HD), Upregulated antisense genes in CM (UP-AS-CM), and downregulated antisense genes in CM (DOWN-AS-CM). Each of the DEG list were analysed against the background of all the genes detected on the array (2-fold above background signal) and statistical significance was assessed using the modified Fisher exact *p*-value of <0.05 (DAVID EASE score) to summarize the molecular functions, biological processes, and cellular components enriched across each disease cohort.

### Library preparation for RNA sequencing

Due to the limited availability of clinical samples and the low yield of high-integrity RNA from patient isolates, RNA extracted from independent biological samples from the same population (uncomplicated and severe patients) was pooled (marked in * in Supplemental Dataset 1). Since the purpose of RNA sequencing was only to validate the transcriptome by a sufficiently robust alternative, this approach ensured that we had sufficient RNA for accurate and reproducible transcriptomic analysis obtained from microarray while still maintaining a representative overview of gene expression, as discussed in this study (Assefa et al., 2020), especially given the critical nature and scarcity of RNA from these disease severities.

Stranded RNA sequencing libraries were prepared using Illumina-compatible NEBNext® Ultra™ II Directional RNA Library Preparation Kit (New England BioLabs, MA, USA) at Genotypic Technology Pvt. Ltd., Bangalore, India. 500 ng of total pooled RNA was taken for mRNA isolation, fragmentation, and priming. Fragmented and primed mRNA was further subjected to first-strand synthesis followed by second-strand synthesis. The double-stranded cDNA was purified using JetSeq Beads (Bioline, Cat # BIO-68031). Purified cDNA was end-repaired, adenylated, and ligated to Illumina multiplex barcode adapters as per NEBNext® Ultra™ II Directional RNA Library Prep protocol, followed by second-strand excision using the USER enzyme at 37 °C for 15 min.

Adapter-ligated cDNA was purified using JetSeq Beads and was subjected to 12 cycles for Indexing- (98 °C for 30 s, cycling (98 °C for 10 s, 65 °C for 75 s) and 65 °C for 5 min) to enrich the adapter-ligated fragments. Final PCR products (sequencing libraries) were purified with JetSeq Beads, followed by a library quality control check. Illumina-compatible sequencing libraries were quantified using a Qubit fluorometer (Thermo Fisher Scientific, MA, USA), and fragment size distribution analysis was performed on an Agilent 2,200 TapeStation. The libraries were paired-end sequenced on an Illumina HiSeq X Ten sequencer (Illumina, San Diego, USA) for 150 cycles following the manufacturer’s instructions.

### RNA sequencing data analysis

Raw reads obtained from the RNA seq experiment were processed using FastQC for quality assessment, and pre-processing was done to remove the adapter sequences and low-quality bases (<q30) using TrimGalore.^2^ The pre-processed high-quality data were first aligned to the human (GRCh38.p13) genome using the splice-aware aligner Hisat2 (with the -rf strandedness parameter; Kim et al., 2019) Host-depleted reads (unaligned to human reference) were aligned against the *Plasmodium falciparum* 3D7 genomev64. Read abundance was calculated using FeatureCounts with strand-setting reversed to capture both sense and NATs (Liao et al., 2014). To increase confidence in antisense capture, we set a threshold of ≥20 antisense reads detected in at least one sequencing pool for downstream analyses. Sequencing (uneven library size/depth) bias among the samples was removed by library normalization using size factor calculation in DESeq2 (Love et al., 2014). DESeq normalized expression values were used to calculate fold change for a given transcript at a log2fold change cutoff ±0.6 to keep it consistent with microarray experimentation. As FeatureCounts would only summarize NATs at the read level, we used StringTie (v2.1.2; Pertea et al., 2015) to assemble transcripts for each gene locus, and the results were compared with the existing genome annotations (v64) using GFFcompare (Pertea and Pertea, 2020) to summarize NATs at the gene level (Feng et al., 2023).

### Library preparation for methylome sequencing

Genome-wide DNA methylome profiling was performed on five samples, two isolates each from cerebral malaria and hepatic dysfunction, and one from the uncomplicated malaria cohort (marked in Supplementary Sheet 1). Due to limited genomic DNA recovery, none of the uncomplicated isolates hybridized to the microarray yielded sufficient DNA for Nanopore sequencing and were therefore replaced with a clinically comparable isolate from the same disease manifestation (uncomplicated isolate NSM1). All samples were processed using identical library preparation, sequencing, and analysis pipelines. Following initial QC, DNA concentration starting from ~1 microgram per sample was barcoded (EXP-NBD104, Oxford Nanopore Technologies) and sequenced as per manufacturer’s instructions (Ligation Sequencing Kit SQK-LSK109, Oxford Nanopore Technologies) on MinION R9.4.1 flow cells (FLO-MN106D), until exhaustion of pores.

### DNA methylation analysis

Raw signals from all samples were base-called with MinKNOW v6.2.8 using the integrated Dorado v7.6.8 basecaller in ‘super-accuracy’ mode (SUP) with 5mC/5hmC modified-base detection in CG contexts. A minimum read Q-score of 10, adapter trimming, and alignment to the *P. falciparum* 3D7 reference genome (v64) were specified during basecalling. Each basecalled BAM file carrying modified-base tags was processed with modKit (v0.6.0)^3^ to generate methylation pileups in BEDmethyl format, tabulating counts of modified and unmodified bases. DNA methylation was quantified in the CpG context using Nanopore sequencing. For each sample, overall modification levels for 5mC and 5hmC were estimated as the proportion of modified reads among all reads covering CpG sites in each sample (∑N_mod/∑(N_mod + N_canonical) with binomial 95% confidence intervals estimated using Wilson intervals. Modified CpGs were annotated to promoter-proximal regions, exons, introns, and intergenic regions using PF3D7 annotation (PlasmoDBv64). Promoters-proximal regions were defined as strand-aware 2-kb upstream regions of annotated transcription start sites. Methylated CpGs overlapping multiple features were assigned to combined categories (e.g., promoter + exon).

### Identification of methylated domains across samples

We used MethyLasso to identify regions with consistent DNA methylation patterns across the entire genome (Balaramane et al., 2024). MethyLasso uses a segmentation approach for long-read data to identify hypomethylated regions, i.e., UMR-DMV (Unmethylated Region-DNA Methylation Valley) and PMD (Partially Methylated Domains) across the genome. The following parameters were specified for running MethyLasso for each isolate independently: –cov 5, –max_distance 2000, –min_width 10. MethyLasso was also run pairwise for each sequenced severe malaria replicate against the uncomplicated replicate (NSM1) to identify DMRs (Differentially Methylated Regions) between uncomplicated and severe samples with a minimum methylation difference of 0.25 (*q* value ≤ 0.05) independently for 5mC and 5hmC.

### Integration of DNA methylation and gene expression

We used a statistical linear regression model to evaluate the relationship between genome-wide CpG methylation and gene expression variability (Tang et al., 2017). To account for gene sequence and the number of CpG sites contributing to each gene-level estimate, CpG density (per kb) was included as a covariate. Thus, the regression model was defined as:

$$\exp_{i}=\beta_{0}+\beta_{1}\cdot {\text{Methy}}_{i}+\beta_{2}\cdot {\mathrm{CpG}}_{i}+\varepsilon_{i}$$

where Exp*_i_* denotes the log-transformed mean of signal intensity for each sense probe of a gene *i,* Methy*_i_* represents the coverage-weighted mean CpG methylation level, CpG*_𝑖_* corresponds to CpG density included in the methylation estimate, and 𝜀_𝑖_ is an error term assumed to follow a normal distribution with mean zero and constant variance. A regression model was fit independently for each of the six biological replicates across three specified genomic intervals: (i) upstream regulatory region (2-kb upstream TSS), (ii) Gene body (inclusive of introns), (iii) downstream regulatory region (2-kb downstream TTS), and the two modification types (5mC and 5hmC). Statistical significance was assessed using two-sided hypothesis tests, and model fit was evaluated using the coefficient of determination (*R*^2^).

Given that 5mC and 5hmC represent alternative modification states of the same cytosine base, we pooled the modification signals at each CpG site and aggregated them within the gene body to calculate the gene-wise methylation frequency for each sample. To ensure robustness of methylation estimates, only genes satisfying the following criteria were included: (i) at least 25% of the annotated gene length was covered by sequencing, and (ii) a minimum of three CpG sites within the gene. The per-gene methylation frequency was compared with the microarray mean signal, and genes were categorized into the top 25% (high) and bottom 25% (low) expression quartiles. Comparisons of aggregated methylation frequencies between high- and low-expression gene groups were performed independently for each sample using the non-parametric Wilcoxon rank-sum test.

## Data Availability

All microarray results have been deposited in the Gene Expression Omnibus database (GSE272963). The RNAseq data investigated in this study are available in IBDC under accession no. INRP000336 (INSDC Study/Bioproject Accession: PRJEB90534). The INDA sample accession IDs are INS0017626 and INS0017627 for complicated and uncomplicated RNA sequencing experiments, respectively. Long-read sequenced methylomes are deposited in IBDC under the accession no. INRP000579 (INSDC Project Accession: PRJEB108452).

## Footnotes

1
https://davidbioinformatics.nih.gov/

2
https://github.com/FelixKrueger/TrimGalore

3
https://github.com/nanoporetech/modkit
