# Supplementary material for: Genome-wide analysis of strand-specific transcription and DNA methylation in Plasmodium falciparum severe malaria
Source: Front Microbiol. 2026 Jun 29;17:1846174. doi: 10.3389/fmicb.2026.1846174 (PMC13357928; doi:10.3389/fmicb.2026.1846174)
Supplement: Supplementary file 1 [file Data_Sheet_1.PDF]

SUPPLEMENTAL FIGURES AND TABLES

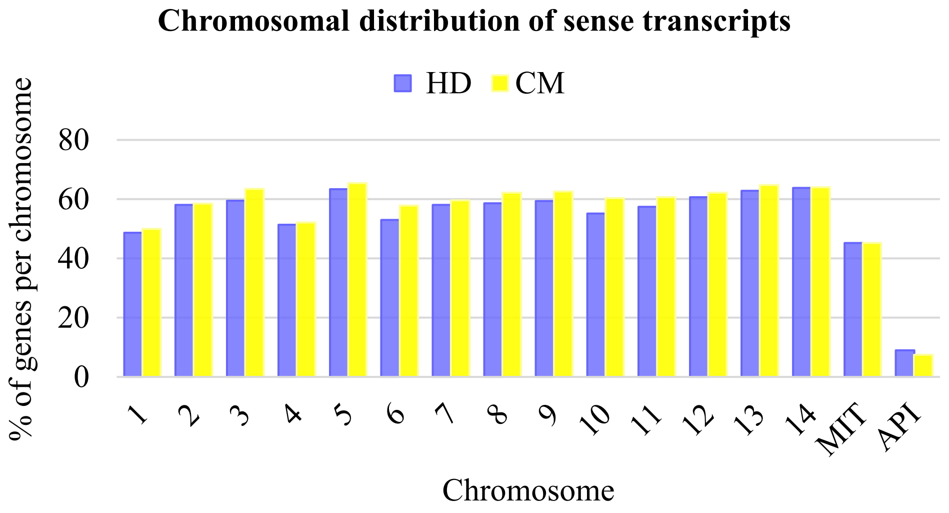

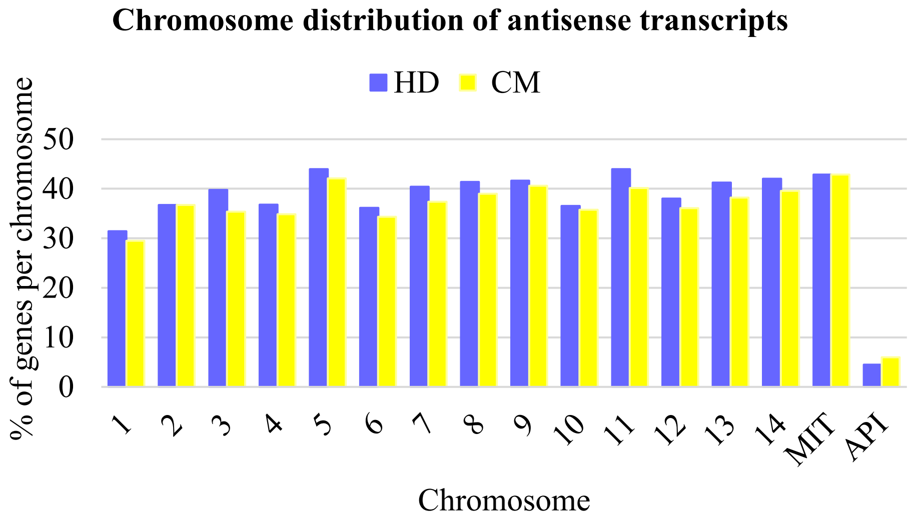

Fig. S1 The figure indicates the percentage of genes with sense and antisense transcription on Y-axis for each chromosome on X-axis.
HD: Hepatic Dysfunction; CM: Cerebral Malaria

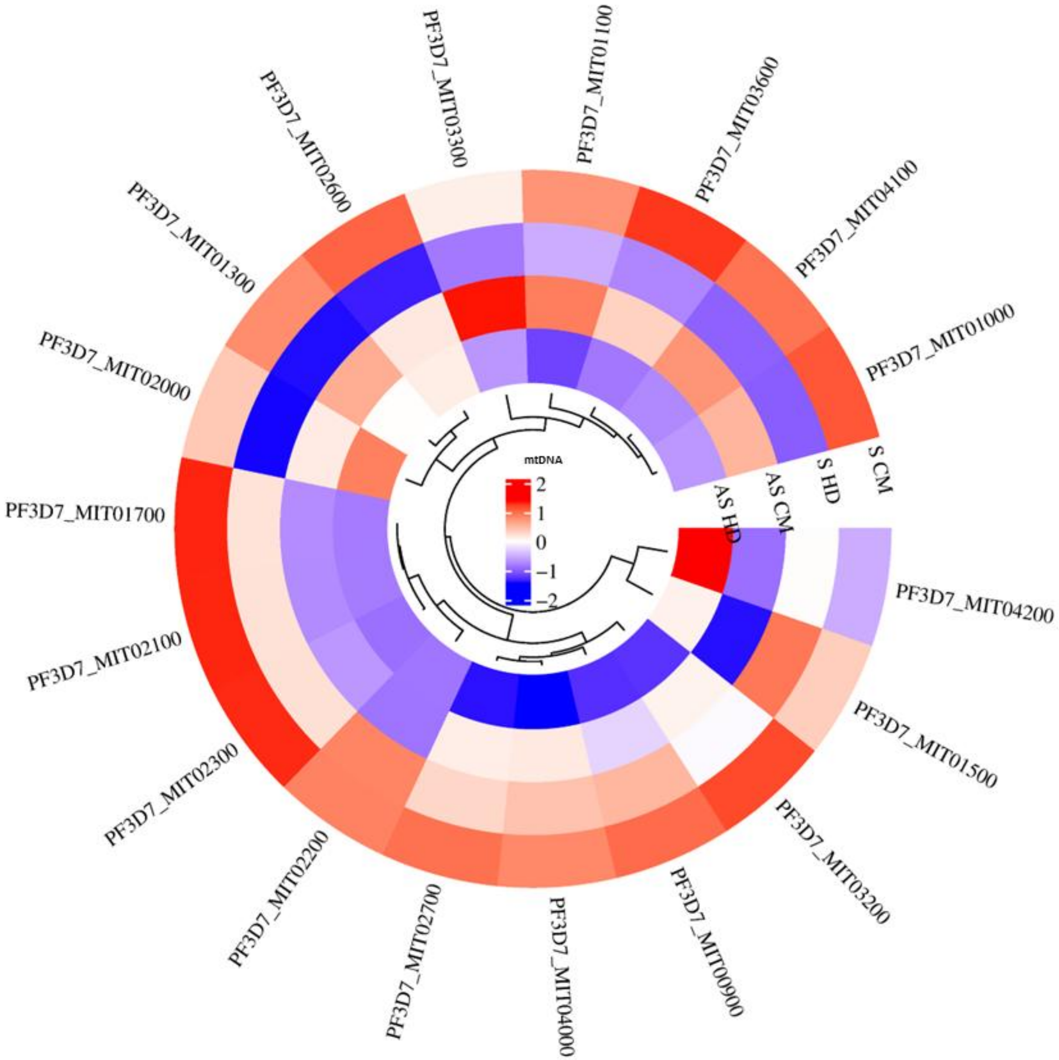

Fig. S2 Four concentric circles representing the log-transformed average expression values of sense and NATs for 18 mitochondrial ORFs detected in microarray experiment with >=3 probes.

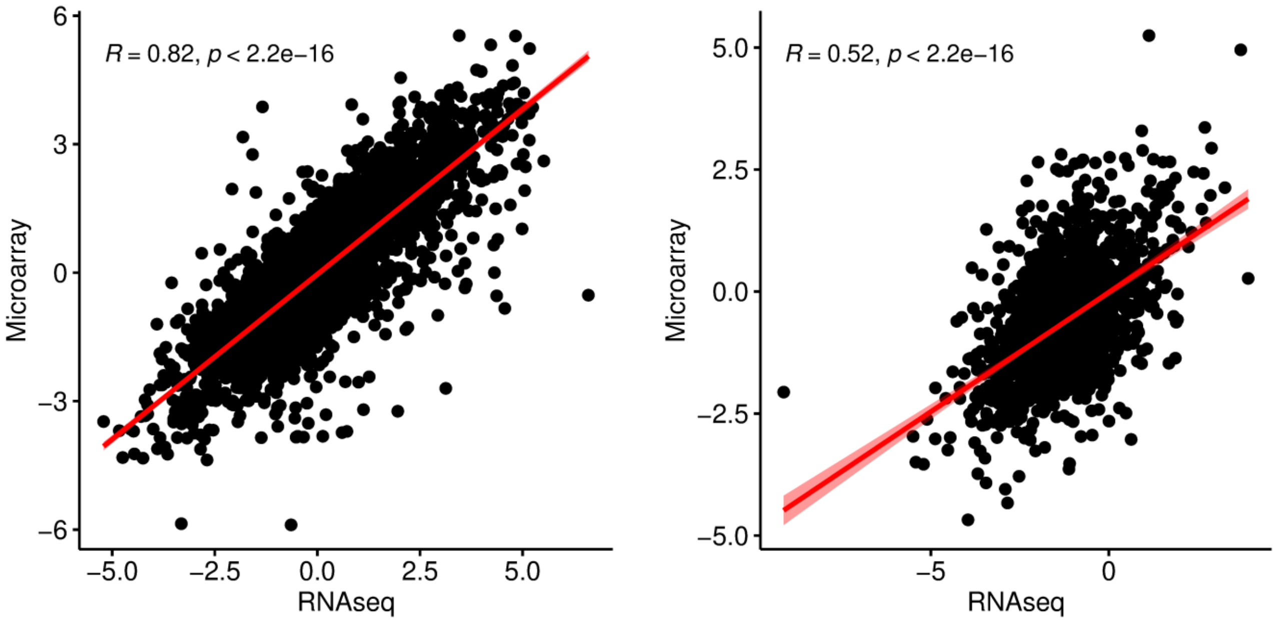

Fig. S3 Correlation of gene expression between RNA-seq and microarray experimentation. Scatter plots show the log2-transformed gene expression values obtained by microarray (y-axis) and RNAseq (x-axis) for each transcript. Each dot represents a gene detected by both platforms. Pearson correlation coefficient (R) : 0.82 for sense transcripts and 0.52 for NATs.

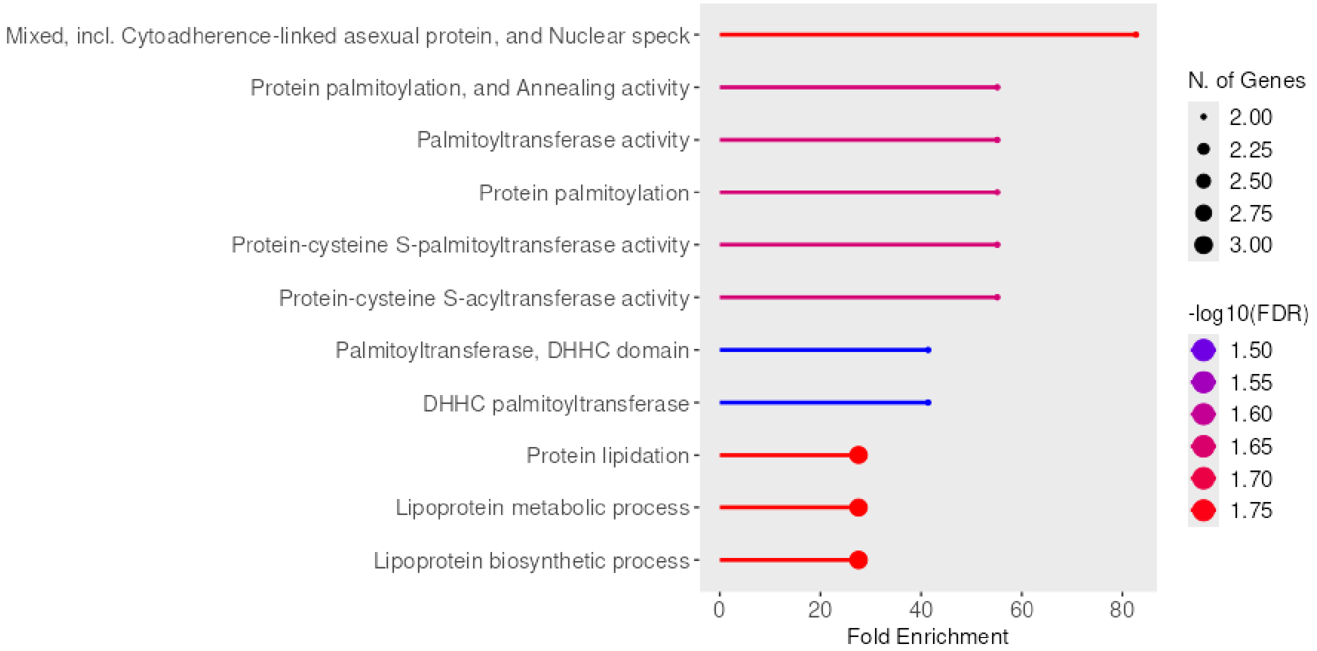

Fig. S4 GO terms enriched for StringTie-assembled antisense transcripts shared across uncomplicated and complicated pools. Enriched terms are shown by fold enrichment, with point size indicating the number of genes and color representing −log10(FDR).

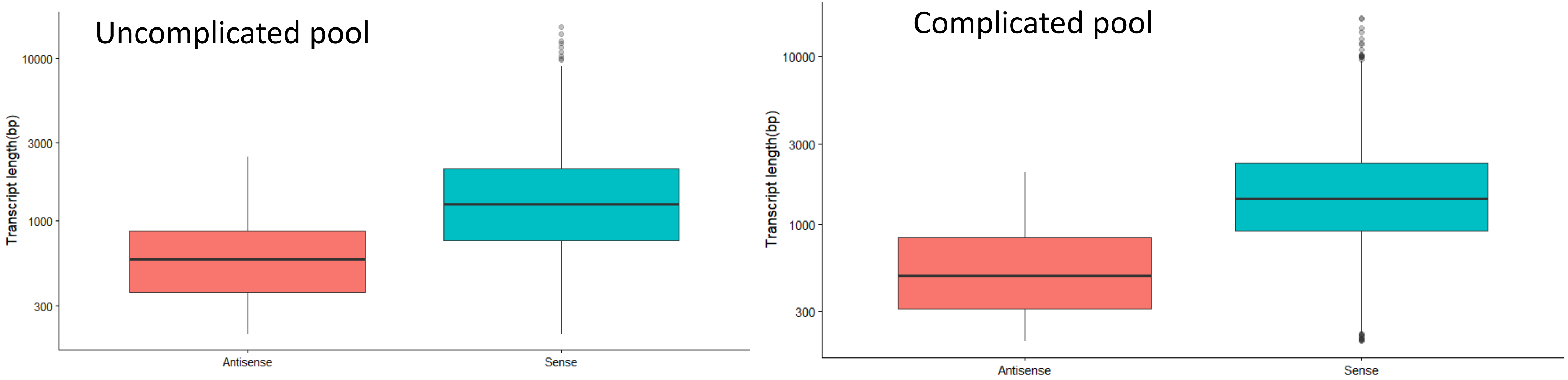

Fig. S5 Comparison of transcript lengths between antisense and sense transcripts assembled using StringTie in uncomplicated and complicated sample pool.

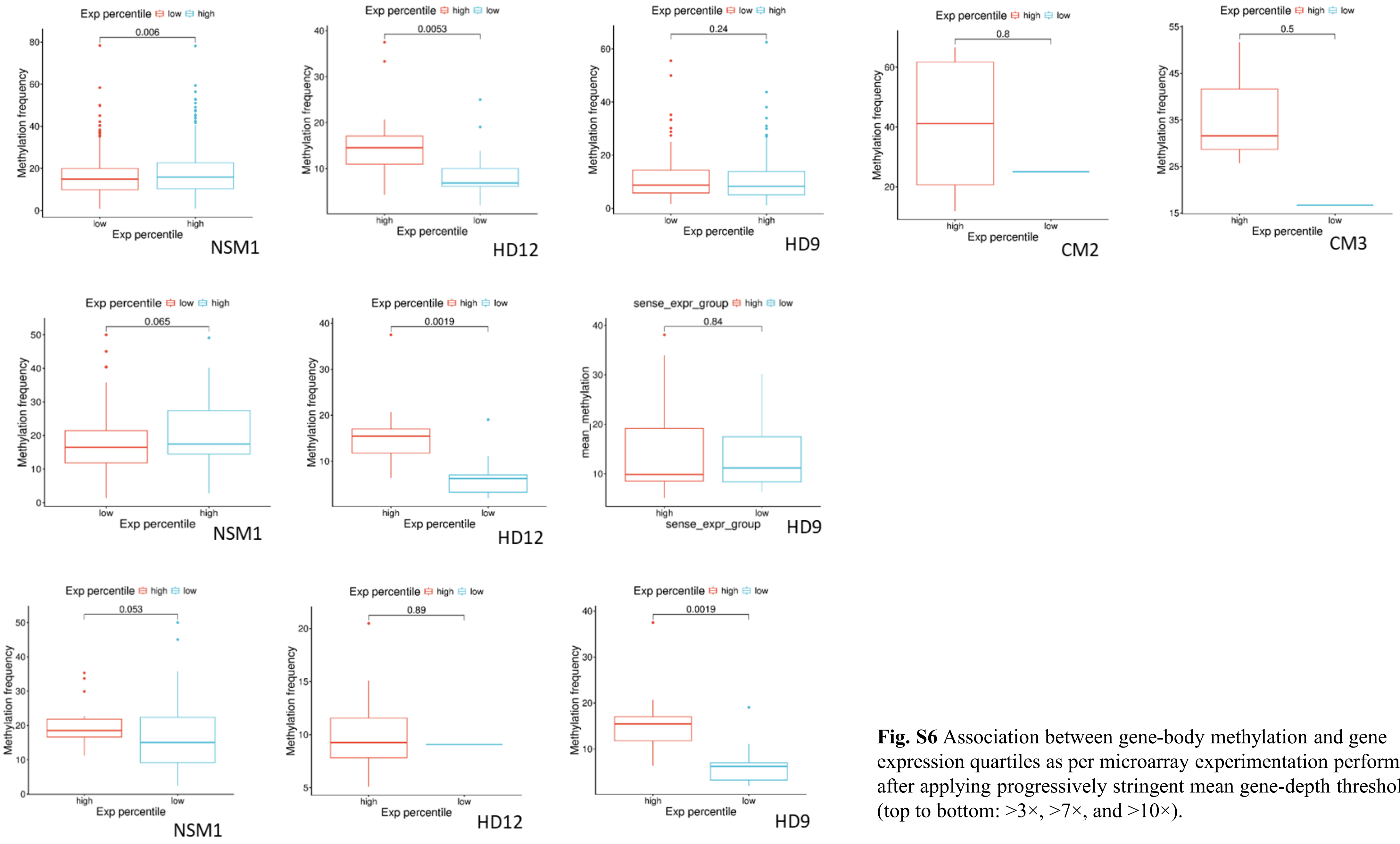

Fig. S6 Association between gene-body methylation and gene expression quartiles as per microarray experimentation performed after applying progressively stringent mean gene-depth thresholds (top to bottom: >3×, >7×, and >10×).

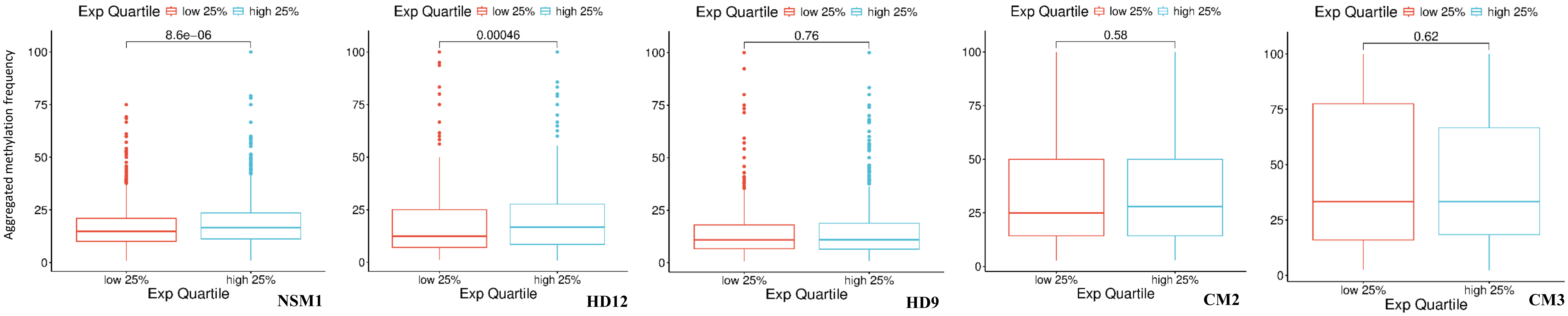

Fig. S7 Aggregated intragenic methylation frequency for genes classified into the lowest 25% (red) and highest 25% (blue) expression quartiles based on TPM values derived from pooled RNA sequencing data, with corresponding p-values indicated above each comparison.

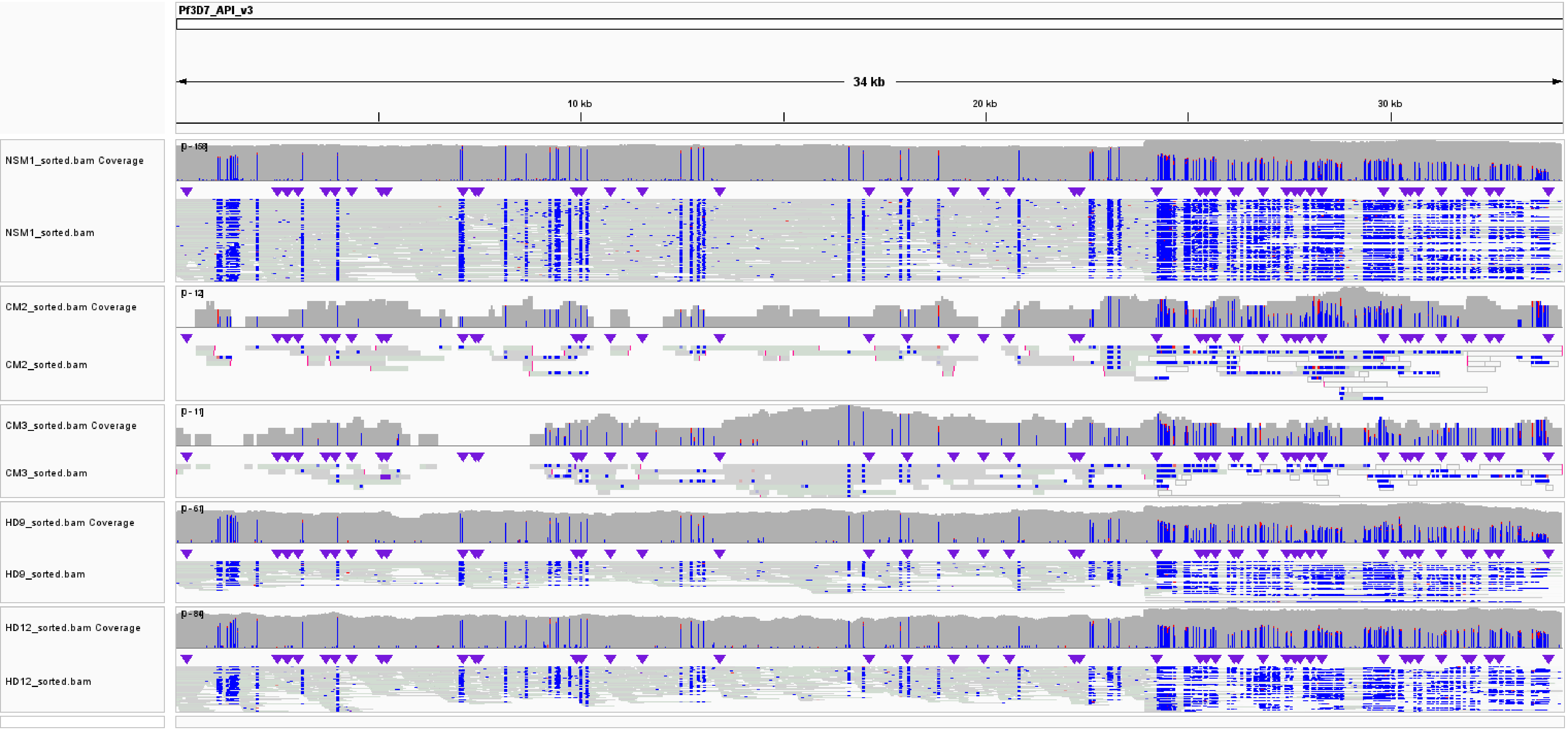

Fig. S8 Integrated Genomics Viewer (IGV) visualization showing modBAM tracks for all the sequenced clinical isolates, aligned to the apicoplast genome of *Plasmodium*. Colored marks in blue represent cytosine base modifications (5mC) detected by Nanopore.

Table S1: Gffcompare-based classification of assembled transcripts in uncomplicated and complicated RNA-seq pools.

Category	Uncomplicated	Complicated pool
Assembled transcripts matching reference annotation	2359	2885
Transcripts with antisense orientation	218	112
Novel/Intergenic transcripts	203	63

Table S2: The table shows the number of genes and the correlation between NAT expression of a reference gene and sense expression of the gene located downstream of the reference gene in a tail-to-head orientation (same strand) and for which the downstream gene is located on the opposite strand (tail-to-tail).

	CM	HD
**Tail-to-Head**		
**No. of gene pairs**	1086	1052
**Pearson correlation**	0.11	0.069
**P value**	0.00042	0.025
**Tail-to-Tail**		
**No. of gene pairs**	1370	1258
**Pearson correlation**	0.05	0.074
**P value**	0.062	0.0088

Table S3: Overlap between 5hmC CpG loci identified in the current study and previously reported 5hmC CpG loci from *Plasmodium falciparum* 3D7 ring and schizont stages (Hammam *et al.*, 2020) [24].

**Samples**	**NSM**	**CM2**	**CM3**	**HD9**	**HD12**
**Ring stage**					
% of overlapping sites between 5hmC CpG loci detected by Hammam et. al., using BS-seq and oxBS-seq, and 5hmC CpG loci identified in the current study	82.44	10.71	6.80	58.81	27.95
% of overlapping methylated sites between 5hmC CpG loci detected by Hammam et. al., using BS-seq and oxBS-seq, and 5hmC CpG loci identified in the current study	31.33	1.06	1.30	9.70	4.05
**Schizont stage**					
% of overlapping sites between 5hmC CpG loci detected by Hammam et. al., using BS-seq and oxBS-seq, and 5hmC CpG loci identified in the current study	83.72	10.85	5.76	60.51	28.92
% of overlapping methylated sites between 5hmC CpG loci detected by Hammam et. al., using BS-seq and oxBS-seq, and 5hmC CpG loci identified in the current study	34.73	1.50	1.01	8.91	3.77
